# Stigma reduces and social support increases engagement in medical care among persons with HIV infection in St. Petersburg, Russia

**DOI:** 10.7448/IAS.17.4.19618

**Published:** 2014-11-02

**Authors:** Jeffrey Kelly, Yuri Amirkhanian, Alexey Yakovlev, Vladimir Musatov, Anastasia Meylakhs, Anna Kuznetsova, Nikolay Chaika

**Affiliations:** 1 Center for AIDS Intervention Research (CAIR), Medical College of Wisconsin, Milwaukee, WI, USA; 2 Department of Medicine, St. Petersburg State University, St. Petersburg, Russian Federation; 3 Interdisciplinary Center for AIDS Research (ICART), Botkin Hospital for Infectious Diseases, St. Petersburg, Russian Federation; 4 Information, St. Petersburg Pasteur Institute, St. Petersburg, Russian Federation

## Abstract

**Introduction:**

The proportion of people living with HIV (PLH) in care and on antiretroviral therapy (ART) in Russia is lower than in Sub-Saharan Africa [1]. This is undoubtedly due to a variety of systems and structural issues related to poor treatment access, linkage and care delivery models. However, little research has explored the reasons that PLH are not in care from their own perspectives. This information can help to guide the development of approaches for improving HIV care engagement in the country.

**Materials and Methods:**

In-depth interviews were undertaken with 80 PLH in St. Petersburg who had never been in HIV medical care, had previously been out of care, or had always been in care. Participants were recruited through online PLH forums and Websites, outreach needle exchange and non-government organisation (NGO) programs, and chain referral. The interviews elicited detailed information about participants’ experiences and circumstances responsible for being out of care, and factors contributing to nonretention in HIV treatment. Verbatim transcriptions of the interviews were coded and analyzed using MAXQDA software to identify emerging themes.

**Results:**

Two types of care engagement barriers most often emerged. Some related to medical services, and others to the family and social environment. The most frequent medical service barriers were poor treatment infrastructure conditions and access; dissatisfaction with quality of services and medical staff; and concerns over confidentiality and HIV status disclosure. Social barriers were fears of potential harm to family relationships, negative consequences if status became known at work, and public stigmatization and myths associated having an HIV+ status. Social support from the PLH community and from family and close friends facilitated care engagement, as did motivation to take care of oneself and one's family. Most participants also described circumstances in which engaging into HIV care was brought about by an urgent issue (opportunistic infections) or was enforced through hospitalization or imprisonment. Trust in one's doctor and simply not wanting to die were also common motives.

**Conclusions:**

Stigma was a major barrier to care engagement, including fear that others would learn of one's HIV+ status, whether at work, in one's family, or in the general community. By contrast, support from family, friends and the PLH community contributed to care engagement.
